# Role of arginine supplementation on muscular metabolism and flesh quality of Pacific white shrimp (*Litopenaeus vannamei*) reared in freshwater

**DOI:** 10.3389/fnut.2022.980188

**Published:** 2022-08-31

**Authors:** Meifeng Li, Hua Wen, Feng Huang, Meili Wu, Lijuan Yu, Ming Jiang, Xing Lu, Juan Tian

**Affiliations:** ^1^Key Laboratory of Freshwater Biodiversity Conservation, The Ministry of Agriculture and Rural Affairs, Yangtze River Fisheries Research Institute, Chinese Academy of Fishery Sciences, Wuhan, China; ^2^Key Laboratory for Animal Nutrition and Feed Science of Hubei Province, Wuhan Polytechnic University, Wuhan, China

**Keywords:** arginine metabolites, hardness, myofiber, nutritional value, shrimp

## Abstract

It is no doubt that the improvement of flesh quality of Pacific white shrimp (*Litopenaeus vannamei*) reared in freshwater contributes to its development potential in aquaculture. In this study, we aimed to explore the effect of arginine supplementation on the flesh quality of *L. vannamei* reared in freshwater and its mechanism. *L. vannamei* were randomly fed with three diets for 56 days, of which arginine level was 10.15 g kg^–1^ (arginine-deficient diet), 21.82 g kg^–1^ (arginine-optimal diet), and 32.46 g kg^–1^ (arginine-excessive diet), respectively. Each diet was randomly assigned to triplicate tanks, and each tank was stocked with 35 shrimps (initial weight: 1.70 ± 0.02 g). Results showed the arginine-optimal diet increased the weight gain, flesh percentage, crude protein and flavor amino acid contents in muscle, and improved the flesh hardness by conversing fast myofibers to slow myofibers, increasing myofiber density and myofibrillar length, and promoting ornithine and collagen synthesis. The arginine-optimal diet influenced the purine metabolic pathway by reducing hypoxanthine, xanthine, and inosine contents. Ornithine, citrulline, and glutamate were identified as the key metabolites affecting flesh quality traits after arginine treatments. Only increasing the levels of dietary arginine did not result in an increase in endogenous creatine synthesis in muscle and hepatopancreas. Overall, the arginine-optimal diet improved the flesh quality traits of *L. vannamei* reared in freshwater due to the enhanced muscular hardness, protein deposition, and flavor, which may be contributing to the transformation of muscle fiber type and increase in protein synthesis by the metabolites of arginine (ornithine, citrulline, and glutamate).

## Introduction

Pacific white shrimp (*Litopenaeus vannamei*) has been recognized as one of the excellent farming species in the world due to its advantages of high-temperature resistance, low-salt resistance, fast growth, strong disease resistance, and high survival rate out of water. It has been cultured in seawater, salt-fresh water, and freshwater for its ability to tolerate the wide salinity from 1 to 40‰ (1). Compared with freshwater-cultured shrimps, marine-cultured shrimps with higher nutritional value have a better flavor and a lighter earthy-musty taste, explaining why their price is higher than freshwater-cultured shrimps (2). However, freshwater aquaculture contributes 77% of the world’s farmed aquatic products (excluding aquatic plants) as measured by edible aquaculture production, providing consumers with cost-effective, accessible, and stable supplies of aquatic products (3). It is no doubt that the improvement of the flesh quality of *L. vannamei* reared in freshwater promotes the development of its aquaculture.

Arginine is an essential amino acid since it plays a crucial physiological role in the growth performance of shrimps (4). Arginine is a precursor for creatine, as it is the only amino acid that can provide the amidino group for creatine synthesis. Our previous study on *L. vannamei* showed that diets supplemented with 8.28 g kg^–1^ creatine enhanced the hardness and chewiness of muscle by improving the diameter and density of myofibers and increasing collagen content (5). Arginine also plays a vital role in producing nitric oxide, polyamine synthesis, inflammation, and innate immune responses (6). Moreover, arginine is the central reserve of high-energy phosphate for the regeneration of ATP in the muscle. Diet is the primary source of arginine for farmed shrimp. The optimal arginine level was 19.60 g kg^–1^ for *L. vannamei* reared in freshwater by two slope broken-line model based on a specific growth rate (SGR) (7). Meanwhile, the optimal arginine in a diet has a positive effect on muscle quality in grass carp (8), finishing pigs (9), and broiler (10). To the best of our knowledge, the relationship between dietary arginine and flesh quality in *L. vannamei* remains largely unresolved.

Consequently, in the current study, three diets containing different arginine levels (deficient, optimal, and excessive) were formulated and hand-fed to juvenile *L. vannamei* for 56 days. To investigate the effect of dietary arginine on growth performance and flesh quality of *L. vannamei* cultured in freshwater and its mechanism, we assessed growth performance, proximate composition, flesh texture, hydroxyproline content, myofiber characteristics, muscular amino acid content, as well as untargeted and targeted metabolomics analyses of muscle in *L. vannamei*. Present study’s findings will provide a deeper understanding of dietary arginine on the flesh quality and arginine metabolism of shrimp and thus eventually provide a novel technology to produce high-quality aquatic products.

## Materials and methods

### Experimental diets and design

Arginine (99.9% in purity) was obtained from Shanghai Macklin Biotechnology Co., Ltd. (Shanghai, China). We selected wheat gluten meal and fishmeal as the primary protein source and chose fish oil and soybean oil as the primary lipid source in the current study. We designed a basal diet including 420 g kg^–1^ protein and 50 g kg^–1^ lipid to fulfill the nutritional requirements of *L. vannamei* (4). We added arginine of 0, 10, and 20 g kg^–1^ to the basal diet, and arginine contents in three diets were analyzed to be 10.15 g kg^–1^ (arginine-deficient diet, LA), 21.82 g kg^–1^ (arginine-optimal diet, MA), and 32.64 g kg^–1^ (arginine-excessive diet, HA). All ingredients were ground into a powder-like condition, sifted, and mixed with oil and arginine. The dough was extruded through a 2-mm diameter template, and then the noodle-like diets were gelatinized at 90°C for 10 min and then dried in a 60°C oven. The feeds were broken into small pellets and stored at −20°C until used. The diets’ raw ingredients, proximate composition, and amino acid profiles are shown in Supplementary Tables 1, 2.

### Feeding management

The feeding trial was conducted at Tongwei Special Aquatic Research Institute (Zhuhai, China). The juvenile *L. vannamei* were purchased from a commercial hatchery (Zhuhai, China). Before the formal feeding trial, the shrimps were stocked in 12 indoor circular tanks (0.70 m in diameter, 0.85 m in height; water volume: 320 L) and fed with the control diet for 2 weeks to acclimate to the experimental conditions. Subsequently, the healthy juvenile shrimps (initial body weight: 1.70 ± 0.02 g) were randomly transferred into nine circular tanks (0.70 m in diameter, 0.85 m in height; water volume: 320 L). Each tank was allocated 35 shrimps. Each experimental diet was randomly assigned to triplicate tanks. The experimental shrimps were hand-fed to apparent satiation four times every day at 7:00, 12:00, 17:00, and 22:00. Uneaten feed and feces were collected after feeding for 2 h. During the feeding period, the water quality was monitored daily, and the results were as follows: dissolved oxygen was >6 mg L^–1^, salinity was 2–3‰, ammonia nitrogen was <0.5 mg L^–1^, and water temperature and pH were maintained at 28.00 ± 2.00 and 7.60–7.8, respectively. The growth trial lasted 56 days.

### Sample collection

After a 56-day feeding, shrimps were deprived of feeds for 24 h. The total number and weight of shrimps in each tank were recorded to calculate survival rate (SR), weight gain rate (WGR), specific growth rate (SGR), and feed conversion ratio (FCR). Next, the body weight of six shrimps per tank was measured, and blood samples were collected using a 1-mL syringe from the pericardial cavity and then centrifuged at 5, 200 g for 15 min at 4°C. Serum samples were separated, transferred into centrifuge tubes of 1.5 mL, and stored at −80°C until analysis. Muscle and hepatopancreas tissues from these six shrimps were collected and weighed to calculate hepatosomatic index (HSI) and flesh percentage (FP). The muscle was cut of similar size and stored at −40°C for texture analysis. Another three shrimps from each tank were sampled to evaluate myofiber ultrastructure and microstructure structure: Approximately 8-mm^3^ muscle from each shrimp was collected in a 10 mL centrifuge tube containing fixative fluid and stored at 4°C for transmission electron microscopy (TEM) analysis. Muscle with size (0.5 cm × 0.5 cm × 0.5 cm) per shrimp was suspended in 4% paraformaldehyde with phosphate buffered saline (PBS) for 24 h and stocked in 70% ethanol for histology. The muscle samples for histology and TEM were taken from the same region from different shrimp across treatment. Muscle and hepatopancreas tissues from another six shrimps per tank were stored at −80°C for gene expression analysis and metabolic profiling. The muscles of the remaining shrimp were collected and stored at −20°C until the determination of collagen contents, amino acid profile, and proximate composition.

### Proximate composition analysis

The proximate composition of muscle and diets were detected by the standard methods (11). The moisture content was dried using a vacuum freeze dryer (Christ Beta 2–4 LD plus LT, Marin Christ Corporation, Osterode, Germany) for 48 h. Crude protein content was determined by an automatic Kjeldahl nitrogen analyzer (Haineng k9840, Shandong Haineng Scientific Instrument Co., Ltd., Dezhou, China) after acid digestion. Crude lipid content was determined by the petroleum-ether extraction method. Ash content was detected at 550°C for 24 h.

### Determination of collagen content

The collagen concentration is calculated by hydroxyproline (Hyp) content. The muscular alkaline-soluble Hyp and alkaline-insoluble Hyp were separated according to the method described by Li et al. (12). In brief, after muscle samples were homogenizing, alkaline-soluble Hyp and alkaline-insoluble Hyp were extracted with sodium hydroxide (0.2 M) and 8 M hydrochloric acid (HCl) under an ice bath, respectively. The determination of Hyp was performed using the method described by Zhang et al. (13). Hydrolytic Hyp content was analyzed by a standard curve.

### Muscular texture properties analysis

Prepared shrimp samples were cooked in boiling water for 1 min, and muscles from the same area were shaped into small cubes (1.0 cm × 1.0 cm × 1.0 cm). Texture parameters, including hardness, springiness, chewiness, cohesiveness, and resilience, were examined using a texture analyzer (model TVT-300XP, Beijing, China) and a cylindrical aluminum probe with a diameter of 50 mm. The measurement parameters were as follows: pre-test tested and post-test speed was set as 2 and 5 mm s^–1^, respectively, and the deformation was 50% of muscle thickness. Each sample was pressed twice, 30 s per. The texture indices were analyzed using a texture analyzer program (version 3.42, Perten Instruments Inc., Hägersten, Sweden).

### Histological properties and ultrastructure analysis

Muscle samples were dehydrated in graded ethanol and then cleared by xylene. Embedded in paraffin, cross-sections and longitudinal sections were sliced (5 μm) and stained with hematoxylin and eosin (H&E) to assess the morphology. The microstructure of muscle was observed using a light microscope (Olympus BX53, Tokyo, Japan). The myofiber characteristics, including myofiber diameter, density, length of the sarcomere, and I-band and A-band, were measured using Image-J Launcher software. Myofiber size is divided into three classes: Classes I, II, and III were categorized according to *d* ≤ 20, 20 < *d* ≤ 50, and *d* > 50, respectively. Class I myofibers were classified as hyperplasia fibers. Class III fibers were categorized as hypertrophic fibers according to the method of Almeida et al. (14). Myofiber density (N/mm^2^) was indicated as a rate of the total number of fibers in the whole area. The myofibrillar structure was conducted with TEM (Hitachi, Ltd., Tokyo, Japan, HT7800) at 80 kV in Wuhan Goodbio Technology Co., Ltd. (Wuhan, Hubei).

### Analyses of hydrolyzed amino acids and free amino acids content

The HAAs were detected by an automatic Amino Acid Analyzer (HITACHI L-8900, Tokyo, Japan). The pretreatment of samples to be tested was as follows: Dried feed and flesh (conducted in vacuum freeze dryer) were transferred into sealed glass tubes and hydrolyzed with 6 M HCl at 110°C for 24 h. Subsequently, the hydrolysate was filtered and diluted to 100 mL with distilled water. The filtrate (2 mL) was taken out and evaporated to dryness at 60°C to remove the HCl in a vacuum dryer for 24 h. Distilled water (2 mL) was added and evaporated to dryness for 24 h again. Then, the sedimentation was dissolved in 8 mL of 0.1 M HCl, which was filtered by a 0.22 μm Millipore membrane, and the supernatant of 1 mL was used for the analysis of HAAs.

The FAAs were measured by an automatic Amino Acid Analyzer (HITACHI L-8900, Tokyo, Japan). The preparation of muscle samples was as follows: the fresh muscle samples were mixed with 10% sulfosalicylic acid and homogenized for 1 min. The homogenate was centrifuged (14, 400 g, 15 min, 4°C) and kept at room temperature for 5 min. After filtering through a 0.22 μm Millipore membrane, the supernatant of 1 mL was used for the FAAs analysis.

### Metabolic profiling

A comprehensive untargeted metabolomics profiling of shrimp muscles was conducted by liquid chromatography and mass spectrometry (LC-MS). Metabolite extraction was conducted as follows: muscle samples of 50 mg were mixed with 800 μL methanol (80%) and then ground for 90 s. After being vortexed (65 Hz, 30 min, 4°C), the samples were centrifuged for 15 min at 4°C with 14, 400 g. Again, the supernatant was collected and centrifuged (4°C, 14, 400 g, 15 min). Dichlorophenylalanine (5 μL) served as the internal standard was added to the supernatant (200 μL) and transferred to an injection vial for LC-MS analysis.

LC-MS analysis and data preprocessing: LC- MS (Waters, UPLC; Thermo, Q Exactive) was performed with an ACQUITY UPLC HSS T3 column (2.1 × 100 mm, 1.8 μm) that was used for the separation of metabolites. Chromatographic separation conditions were as follows: the column temperature was 40°C, and the flow rate was 0.30 mL min^–1^. The mobile phase consisted of A: water +0.05% formic acid and B: acetonitrile. The prepared sample was placed in an automatic sampler at 4°C with an injection volume of 1 μL during the whole analysis process.

Metabolites were detected by electrospray ionization (ESI) positive and negative ion patterns. Detecting parameters for ESI+ mode were as follows: Heater temperature 300°C; sheath gas flow rate, 45 arb; aux gas flow rate, 15 arb; sweep gas flow rate, 1 arb; spray voltage, 3.0 kV; capillary temperature, 350°C; S-lens RF level, 30%. For ESI- model: Heater temperature 300°C, Sheath Gas Flow rate, 45 arb; Aux Gas Flow Rate, 15 arb; Sweep Gas Flow Rate, 1 arb; spray voltage, 3.2 kV; Capillary temperature, 350°C; S-Lens RF Level, 60%.

The raw data were extracted and preprocessed using Compound Discoverer software (Thermo). The data were normalized and post-edited in Excel 2010 software and finally edited into a two-dimensional data matrix. After the data is preprocessed, orthogonal partial least squares discriminant analysis (OPLS-DA) is performed. The variable importance in the projection (VIP) scores from OPLS-DA are calculated for each component. Differential metabolites (DFMs) between two groups were selected according to the following screening conditions: VIP > 1 and Student’s *T*-test with *P*-value < 0.05. Then, the disturbed metabolic pathways of differential metabolites were analyzed by searching the online Kyoto Encyclopedia of Genes and Genomes (KEGG) database.

### Metabolites analysis of arginine

There are nine representative metabolites of arginine in muscle and hepatopancreas, including citrulline, adenosine, adenine, inosine, glycocyamine, creatine, creatinine, and ornithine fumaric acid which were selected for quantitative analysis using an ultrahigh-performance LC/MS with a triple quadrupole mass spectrometry (UPLC-QQQ-MS) approach. Above samples of 50 mg were weighed and premixed with 600 μL acetonitrile (80%). After putting under vortex movement for 2 min, the samples were ground for 90 s at 60 Hz, and then conducted an ultrasound for 30 min at 4°C. The extracts were centrifuged at 14, 000 g for 15 min at 4°C. Finally, the supernatant of 500 μL was transferred to vials for determining the contents of the target components through the LC-MS/MS system.

Analyses of interest were separated from prepared samples using Waters Acquity UPLC (Ultra performance liquid chromatography) equipped with an Acquity UPLC HSS T3 column (1.8 μm, 2.1 mm × 100 mm) and its temperature was 40°C at a flow rate of 0.25 mL min^–1^. Mobile phase A consisted of 0.10% aqueous formic acid, and mobile phase B was composed of methanol. The separated compounds were identified using AB SCIEX 5500 QQQ–MS (mass spectrometry). MS parameters were set as follow: (Curtain Gas): 35 arb; (Collision GAS): 7 arb; (IonSpray voltage): 4.5 kV; (IonSpray voltage): 4.5 kV; (Temperature): 450°C; (IonSource Gas1): 55 arb; (IonSource Gas2): 55 arb. Eventually, a prepared standard solution was added to the vial, and quantification was performed under multiple reaction monitoring (MRM) mode. MultiQuant software for integration and standard curve for content calculation. The standard solution was diluted with acetonitrile (80%) to prepare a series of appropriate concentrations for establishing the calibration curve. The calibration curve was plotted according to the peak areas of the standards at different concentrations and corresponding concentrations.

### Gene expressions analysis in muscle

Total RNA was isolated from muscle using TRIzol reagent (Life Technologies, Carlsbad, CA, United States), and the cDNA was generated following the instructions of the PrimeScript^®^ RT reagent kit (Takara Biotech, Dalian, China). SYBR^®^ Premix Ex Taq™ (Takara Biotech, Dalian, China) was utilized to quantify the expressions of MyHCs gene (*sMyHC1*, *sMyHC2*, *sMyHC5*, *sMyHC6a*, and *sMyHC15*), and elongation factor 1-alpha1 (*EF-1α1*) acted as a reference gene. Real-time polymerase chain reaction (Real-time PCR) was conducted on a quantitative thermal cycler (Light Cycler 480II, Roche) and was performed as in the previous study (5). Primer sequences were presented in Supplementary Table 3 and were synthesized by Sangon Biotech Co., Ltd. (Shanghai, China). Relative quantification of transcript expression was calculated using the 2^–ΔΔ*CT*^ method, and the relative mRNA expressions of the LA group was set as standard.

### Serum urea nitrogen analysis

Each serum sample (200 μL) was transferred into a sample cup, and the concentrations of urea nitrogen in the serum were quantified by urease/GLDH method using an automatic biochemistry analyzer (CHEMIX-800, Sysmex Corporation, Kobe, Japan). All kits were purchased from Sysmex Corporation.

### Calculation and statistical analysis

All data were presented as mean ± S.D. (standard deviation). The homogeneity of variances and normality of the data was evaluated prior to statistical analysis, which included proximate composition, flesh texture, hydroxyproline content, myofiber characteristics, muscular amino acid content, metabolites of arginine contents. All data were analyzed by one-way analysis of variance (ANOVA) and Tukey’s multiple comparison tests using the SPSS 22.0 (SPSS, United States) for Windows. The significant difference was set at *P* < 0.05.

## Results

### Growth performance

The growth performance of *L. vannamei* is provided in Table 1. Shrimps fed with the MA diet had the highest final body weight (FBW), SGR, WGR, flesh percentage (FP) and survival rate (SR), which were significantly higher than those with the LA and HA diets (*P* < 0.05). The highest hepatopancreas indexes (HSI) value occurred in the HA group. Feed conversion ratio (FCR) in the MA group was significantly lower than that in the LA and HA groups (*P* < 0.05).

**TABLE 1:** The growth performance of *Litopenaeus vannamei* after dietary arginine treatment for 56 days.

Indexes	Diets^9^		
	LA	MA	HA
IBW^1^ (g)	1.76 ± 0.10	1.71 ± 0.02	1.79 ± 0.06
FBW^2^ (g)	10.65 ± 0.10^b^	10.68 ± 0.21^b^	10.18 ± 0.15^a^
WGR^3^ (%)	508.20 ± 15.13^ab^	524.53 ± 8.48^b^	469.81 ± 23.71^a^
SGR^4^ (% d^–1^)	3.22 ± 0.03^b^	3.27 ± 0.02^b^	3.10 ± 0.01^a^
FCR^5^	1.15 ± 0.03^b^	1.06 ± 0.02^a^	1.17 ± 0.01^b^
HSI^6^ (%)	3.16 ± 0.29^a^	3.23 ± 0.25^ab^	3.47 ± 0.21^b^
FP^7^ (%)	47.40 ± 2.81^ab^	49.61 ± 1.89^b^	45.42 ± 43.32^a^
SR^8^ (%)	93.42 ± 1.51^b^	96.22 ± 1.13^b^	74.28 ± 0.62^a^

^1^IBW, initial body weight.

^2^FBW, final body weight.

^3^WGR (weight gain rate, %) = 100 × [(final body weight)–(initial body weight)]/initial body weight.

^4^SGR (specific growth rate, % d^–1^) = 100 × ln (final body weight/initial body weight)/days.

^5^FCR (feed conversion ratio) = dry feed consumed/(final biomass - initial biomass + dead fish).

^6^HSI (hepatosomatic index, %) = 100 × (hepatopancreas weight/the whole body weight).

^7^FP (flesh percentage, %) = 100 × (muscle weight/whole body weight).

^8^SR (survival rate,%) = 100 × (final number of shrimp/initial number of shrimp).

^9^LA, the arginine-deficient diet; MA, the arginine-optimal diet; HA, the arginine-excessive diet.

^10^Different letters are significantly different (*P* < 0.05).

### Muscular texture analysis

As shown in Table 2, the LA group had the lowest hardness and chewiness of muscle than other groups (*P* < 0.05). No significant difference occurred in parameters including springiness, resilience, and cohesiveness among all groups (*P* > 0.05).

**TABLE 2:** The muscular textural properties, proximate composition, and collagen contents of *L. vannamei* after dietary arginine treatment for 56 days.

Indexes	Diets^3^		
	LA	MA	HA
**Texture properties**			
Hardness (gf)	1280.29 ± 36.14^a^	1386.43 ± 40.17^b^	1506.43 ± 65.02^c^
Springiness	0.58 ± 0.04	0.59 ± 0.03	0.58 ± 0.04
Chewiness (gf)	407.62 ± 17.70^a^	461.81 ± 20.53^b^	487.37 ± 19.10^b^
Resilience	0.29 ± 0.02	0.28 ± 0.03	0.26 ± 0.02
Cohesiveness	0.57 ± 0.04	0.56 ± 0.04	0.55 ± 0.05
**Proximate composition (g kg^–1^)**			
Moisture	784.82 ± 4.55	781.73 ± 3.58	784.44 ± 2.36
Crude protein	188.35 ± 3.94^a^	197.43 ± 3.06^b^	193.53 ± 2.74^ab^
Crude lipid	5.14 ± 0.17	5.53 ± 0.31	5.18 ± 0.18
Ash	13.22 ± 0.22	13.65 ± 0.29	13.41 ± 0.24
Protein/moisture^1^	0.240 ± 0.006^a^	0.253 ± 0.003^b^	0.247 ± 0.003^ab^
**Hyp (g kg^–1^)**			
Alkaline-soluble Hyp^2^	0.25 ± 0.03^a^	0.40 ± 0.04^b^	0.40 ± 0.04^b^
Alkaline-insoluble Hyp	0.39 ± 0.04^a^	0.54 ± 0.04^b^	0.76 ± 0.03^c^
Total Hyp	0.65 ± 0.04^a^	0.94 ± 0.07^b^	1.16 ± 0.05^c^

^1^Protein/moisture, protein content/moisture content.

^2^ Hyp, hydroxyproline.

^3^LA, the arginine-deficient diet; MA, the arginine-optimal diet; HA, the arginine-excessive diet.

### Proximate composition and collagen content in the flesh

As displayed in Table 2, there were no significant differences in the contents of moisture, crude lipid, and ash in muscle among all treatments (*P* > 0.05). The crude protein content and the protein-moisture ratio of muscle in the MA group were significantly higher than those in the LA group (*P* < 0.05). The LA group had the lowest alkaline-soluble Hyp, alkaline-insoluble Hyp, and total Hyp content.

### Muscular hydrolyzed amino acids profiles

The muscular amino acid composition is presented in Table 3. The contents of aspartate, lysine, and arginine in the LA group were significantly lower than those in the MA and HA groups (*P* < 0.05). As compared to the LA group, the contents of valine, methionine, isoleucine, total amino acids (TAAs), and total essential amino acids (EAAs) were significantly higher in the MA group (*P* < 0.05).

**TABLE 3:** Free amino acid (mg kg^–1^) and hydrolyzed amino acid (g kg^–1^) contents in the muscle of *L. vannamei* after dietary arginine treatment for 56 days.

Amino acids	LA		MA		HA	
	FAA	HAA	FAA	HAA	FAA	HAA
**Essential amino acids**						
Arg	4957.78 ± 87.01^a^	16.09 ± 0.81^a^	5555.73 ± 98.88^b^	18.09 ± 0.88^b^	5039.00 ± 154.58^a^	18.34 ± 0.68^b^
His	112.57 ± 7.14^a^	3.19 ± 0.23	151.32 ± 8.33^b^	3.46 ± 0.18	201.43 ± 5.63^c^	3.52 ± 0.17
Ile	34.10 ± 1.76^a^	6.12 ± 0.54^a^	61.57 ± 2.06^c^	7.59 ± 0.25^b^	51.43 ± 2.99^b^	6.94 ± 0.66^ab^
Leu	76.46 ± 2.09^a^	11.21 ± 1.06	84.84 ± 2.42^b^	13.00 ± 0.45	104.21 ± 4.52^c^	12.41 ± 0.79
Lys	253.22 ± 15.70^a^	11.86 ± 0.99^a^	294.71 ± 22.29^b^	13.74 ± 0.49^b^	271.12 ± 8.65^ab^	13.77 ± 0.47^b^
Met	63.15 ± 3.11	5.02 ± 0.47^a^	66.74 ± 1.27	6.25 ± 0.10^b^	65.50 ± 4.70	5.36 ± 0.52^ab^
Phe	50.73 ± 2.04^a^	6.71 ± 0.59	54.93 ± 2.10^a^	7.30 ± 0.23	78.71 ± 4.22^b^	7.00 ± 0.40
Thr	30.16 ± 2.42^a^	5.44 ± 0.39	30.38 ± 2.16^a^	6.05 ± 0.25	46.84 ± 2.74^b^	6.03 ± 0.22
Val	106.96 ± 4.44	6.48 ± 0.55^a^	117.77 ± 3.01	7.68 ± 0.28^b^	115.71 ± 6.92	7.32 ± 0.39^ab^
**Non-essential amino acids**						
Ala	2292.52 ± 45.92^b^	9.09 ± 0.88	2471.67 ± 63.24^c^	11.62 ± 0.65	2038.96 ± 48.15^a^	11.28 ± 0.08
Asp	248.52 ± 12.95^a^	14.22 ± 1.08^a^	337.83 ± 39.97^b^	16.47 ± 0.87^b^	450.32 ± 18.75^c^	16.49 ± 0.39^b^
Glu	669.79 ± 50.95^a^	24.62 ± 1.63	825.89 ± 29.32^b^	26.95 ± 0.88	1137.47 ± 12.79^c^	26.98 ± 0.68
Gly	3746.6 ± 178.85^b^	13.14 ± 1.27	3724.87 ± 96.87^b^	14.44 ± 0.70	3080.90 ± 120.76^a^	14.02 ± 0.54
Pro	2376.83 ± 212.75^a^	9.12 ± 0.70	2507.26 ± 177.43^a^	9.11 ± 0.33	3204.81 ± 169.27^b^	9.12 ± 0.37
Ser	93.49 ± 7.10^a^	5.94 ± 0.44	128.90 ± 5.96^b^	6.14 ± 0.19	112.98 ± 6.20^b^	6.14 ± 0.19
Tyr	80.30 ± 2.06^a^	6.26 ± 0.29	133.34 ± 1.50^b^	7.22 ± 0.19	133.35 ± 1.88^b^	6.86 ± 0.53
ΣFAA	6957.44 ± 118.09^a^	–	7360.26 ± 26.50^b^	–	6707.66 ± 154.48^a^	–
ΣEAA	5685.12 ± 100.77^a^	72.13 ± 4.61^a^	6417.99 ± 97.43^b^	83.16 ± 2.00^b^	5973.96 ± 162.20^a^	80.69 ± 4.67^ab^
ΣNEAA	9508.05 ± 270.53	82.39 ± 6.37	10129.77 ± 174.15	91.95 ± 3.47	10158.80 ± 318.17	90.88 ± 2.41
TAAs	15193.17 ± 335.37^a^	154.52 ± 9.32^a^	16547.76 ± 135.07^b^	175.10 ± 5.33^b^	16132.76 ± 354.27^b^	171.57 ± 6.66^ab^

FAA, free amino acid.

HAA, hydrolyzed amino acid.

ΣFAA, Flavor amino acids.

ΣEAA, total essential amino acids.

ΣNEAA, total non-essential amino acids.

TAAs, total amino acids.

LA, the arginine-deficient diet; MA, the arginine-optimal diet; HA, the arginine-excessive diet.

### Muscular free amino acids profiles

The FAAs contents of the flesh are displayed in Table 3. Significant higher threonine, phenylalanine, leucine, histidine, aspartate, glutamate, and proline contents were found in the HA group (*P* < 0.05). The MA group had the highest contents of isoleucine, arginine, alanine, Total EAAs, and total FAAs (*P* < 0.05). TAAs in the LA group were significantly lower than that in other groups. The contents of muscular serine and tryptophan in the LA group were significantly lower than that in the MA and HA groups (*P* < 0.05).

### Histological properties and ultrastructure analysis

**FIGURE 1:**
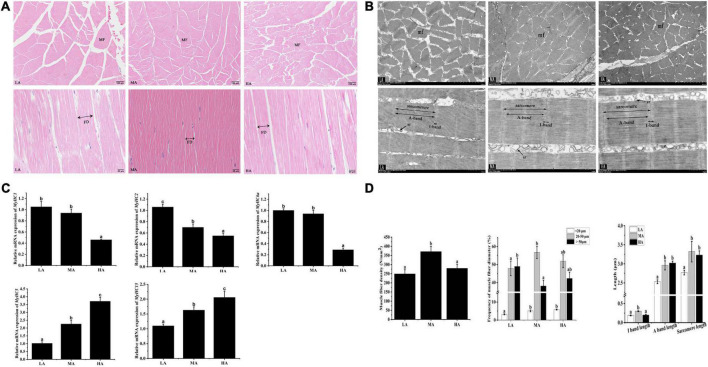
The morphology of myofiber of *Litopenaeus vannamei* after dietary arginine treatment for 56 days. **(A)** Myofiber microstructure of cross-section (Magnification 100×) and longitudinal section (Magnification 400×). **(B)** Transmission electron microscope of cross-section and longitudinal section at a magnification of 3,000× and 8,000×, respectively. **(C)** The density and the lengths of diameter, I band, A band, and sarcomere in myofiber of *L. vannamei*. **(D)** The relative mRNA expression of MyHCs in *L. vannamei*. MF, myofiber. mf, myofibril. FD, myofiber diameter. sr, sarcoplasmic reticulum. sMyHC1, sMyHC2, and sMyHC6a are mainly expressed in fast myofibers. sMyHC5 and sMyHC15 are mainly expressed in slow myofibers. LA, the arginine-deficient diet; MA, the arginine-optimal diet; HA, the arginine-excessive diet.

The effect of arginine on the muscle morphology of *L. vannamei* is shown in Figure 1A. The shape of myofiber exhibited irregular polygons, and the abundant connective tissue separates myofibers. The ultrastructure of myofiber is revealed in Figure 1B. Myofiber diameters and myofiber density are shown in Figure 1C. The muscle density of the MA group was significantly higher than that in the LA group (*P* < 0.05) but had no significant difference compared to the HA group (*P* > 0.05). The percentage of small-sized fiber (<20 μm) in the MA and HA groups was significantly higher than that in the LA group (*P* < 0.05). The highest percentage of fibers of 20–50 μm and the lowest percentage of large diameter fibers (>50 μm) have occurred in the LA group. The lengths of dark areas (A band), light areas (I band), and sarcomere as shown in Figure 1C. The A band length and sarcomeres length (SL) in the LA group significantly decreased compared to that in the MA and HA groups (*P* < 0.05). The I band length in the MA group significantly increased than that in other groups (*P* < 0.05).

### Gene expression

Shrimps fed diets containing different arginine showed different expressions of muscle-related genes (Figure 1D). The fast-type muscle relative gene expressions (*sMyHC1*, *sMyHC2*, and *sMyHC6a*) in the HA group were significantly higher than that in other groups. The expressions of slow-type muscle relative gene (*sMyHC5* and *sMyHC15*) in the LA group were significantly lower than that in the MA and HA groups (*P* < 0.05).

### Metabolomics analysis of metabolites in muscle after graded arginine treatments

**FIGURE 2:**
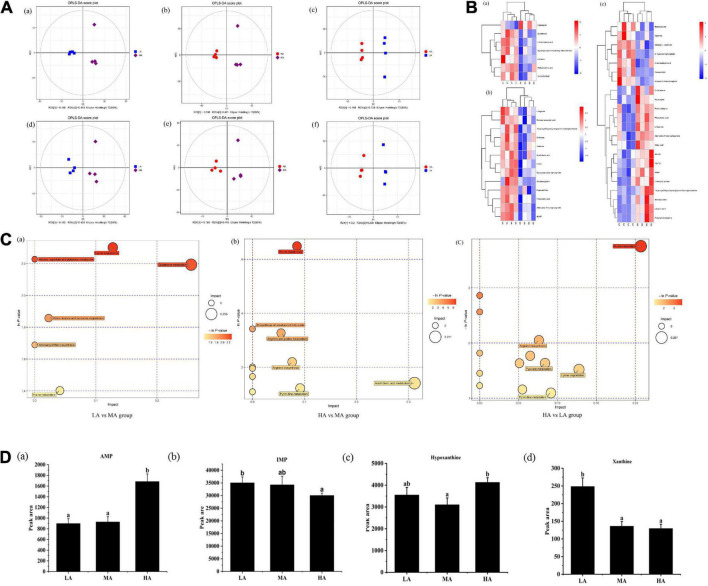
Untargeted metabolomics profiling of muscle in *L. vannamei* after dietary arginine treatment for 56 days. **(A)** OPLS-DA score plot of different comparison groups in ESI+ (a–c) and ESI– (d–f) model. **(B)** Heatmap of differential metabolites in different comparative groups. (a) Between LA and MA groups; (b) between HA and MA groups; (c) between LA and HA groups. **(C)** Metabolome view map of relevant metabolic pathways for change (a) between LA and MA groups; (b) between HA and MA groups; (c) between HA and LA groups. **(D)** Peak areas (× 106) of four flavoring substances involved in purine metabolism were detected based on muscle non-targeted metabolomics. (a) AMP; (b) IMP; (a) hypoxanthine; (a) xanthine. LA, the arginine-deficient diet; MA, the arginine-optimal diet; HA, the arginine-excessive diet.

Untargeted metabolomic analysis of muscle based on OPLS-DA analysis indicated that metabolic patterns changed after *L. vannamei* were fed diets containing different arginine levels. The score plots of OPLS-DA between different groups are depicted in Figure 2A. The cluster separations are acceptable in different groups under positive and negative models.

Heat maps based on the judgment criteria (VIP values > 1, *P* < 0.05) showed the relationships and relative abundances of DFMs between different groups (Figure 2B). The DFMs were analyzed using the MetaboAnalyst platform. The essential metabolic pathways relating to the DFMs are presented in Figure 2C. The plot revealed that purine metabolism might be the main differential pathway among the treatments based on enrichment and metabolic pathway analyses. Analysis of the purine metabolic pathway revealed that there existed four differential metabolites: adenosine monophosphate (AMP), inosine monophosphate (IMP), hypoxanthine, and xanthine, were concentrated in the purine metabolism pathway (Figure 2D). Muscular AMP relative content reached the highest value in the HA group (*P* < 0.05). The IMP relative content in the muscles of the HA group was significantly lower than that of the LA group (*P* < 0.05). The lowest hypoxanthine relative content in the muscle occurred in the MA group (*P* < 0.05). The xanthine relative content in the muscle of the LA group was significantly higher than other groups (*P* < 0.05).

### The contents of metabolites of arginine in muscle, hepatopancreas, and serum

The concentration of nine metabolites related to arginine metabolism (adenosine, adenine, inosine, citrulline, glycocyamine, creatine, creatinine, ornithine, and fumaric acid) in the muscle and hepatopancreas are listed in Table 4. In hepatopancreas, the concentrations of citrulline, adenine, inosine, glycocyamine, creatine, ornithine, and fumaric acid showed no significant difference among all treatments (*P* > 0.05). The contents of adenosine and creatinine in the hepatopancreas in the LA group were lower than that in other groups (*P* < 0.05).

**TABLE 4:** Relative metabolites of arginine contents in the hepatopancreas, muscle, and serum of *L. vannamei* after dietary arginine treatment.

Indexes	LA	MA	HA
**Hepatopancreas (mg kg^–1^)**			
Adenine	1.02 ± 0.07	1.11 ± 0.06	1.03 ± 0.04
Adenosine	2.55 ± 0.17^a^	3.77 ± 0.58^b^	3.85 ± 0.31^b^
Citrulline	7.66 ± 0.21	8.04 ± 0.13	8.07 ± 0.42
Creatine	0.16 ± 0.01	0.17 ± 0.01	0.17 ± 0.01
Creatinine	1.72 ± 0.05^a^	2.38 ± 0.36^ab^	2.60 ± 0.44^b^
Fumaric acid	3.60 ± 0.38	4.14 ± 0.20	4.14 ± 0.25
Glycocyamine	0.88 ± 0.08	0.92 ± 0.05	0.82 ± 0.05
Inosine	3638.66 ± 360.42	3643.60 ± 345.87	3669.17 ± 339.97
Ornithine	60.00 ± 2.44	63.33 ± 1.55	58.39 ± 2.26
**Muscle (mg kg^–1^)**			
Adenine	0.48 ± 0.02	0.50 ± 0.05	0.46 ± 0.02
Adenosine	3.10 ± 0.21^a^	3.75 ± 0.56^ab^	4.40 ± 0.43^b^
Citrulline	10.45 ± 0.37^a^	11.51 ± 0.45^a^	13.96 ± 0.64^b^
Creatine	1.15 ± 0.04	1.23 ± 0.08	1.29 ± 0.08
Creatinine	1.71 ± 0.10^a^	2.18 ± 0.08^b^	3.15 ± 0.20^c^
Fumaric acid	44.92 ± 2.07^c^	39.84 ± 2.56^b^	26.62 ± 1.17^a^
Glycocyamine	0.83 ± 0.05	0.81 ± 0.05	0.83 ± 0.02
Inosine	542.60 ± 32.50^c^	369.61 ± 25.57^b^	302.78 ± 18.94^a^
Ornithine	43.34 ± 1.06^a^	43.57 ± 1.03^a^	69.40 ± 2.69^b^
**Serum (mmol L^–1^)**			
Urea nitrogen	6.33 ± 0.25^a^	6.93 ± 0.32^ab^	7.13 ± 0.18^b^

LA, the arginine-deficient diet; MA, the arginine-optimal diet; HA, the arginine-excessive diet.

Muscular citrulline content reached the highest value in shrimps fed with the HA diet. The lowest contents of muscular inosine and fumaric acid were detected in the HA group. Muscular adenosine, creatinine, and ornithine contents in the HA group were significantly higher than in the LA and MA groups (*P* < 0.05). Adenine, glycocyamine, and creatine contents in the muscle showed no significant difference among all groups (*P* > 0.05). Serum urea nitrogen content increased with the increasing dietary arginine levels.

## Discussion

The current study showed that *L. vannamei* reared in freshwater fed a diet containing optimum arginine level had a good growth performance, which agreed with other crustaceans, such as juvenile Kuruma shrimp (*Marsupenaeus japonicus*) (15), *Penaeus monodon* (16). Notably, the present study indicated that excessive arginine in the diet led to a decline in growth and survival rate, together with a higher FCR of *L. vannamei* cultured in freshwater. Maybe excessive arginine disturbed physiological metabolism and consequently induced toxic effects and stress in the shrimps, leading to extra-energy expenditure used for deamination and excretion (4, 17, 18).

It is the key of this research to reveal the relationship between arginine metabolites and flesh quality, which will provide new insight into the design of effective nutritional strategies for improving the flesh quality of *L. vannamei*. We measured the metabolites of arginine using untargeted and targeted metabolomics. The enrichment analysis and metabolic pathway analysis showed that the differential metabolic pathways were “purine metabolism” pathway, and dietary arginine levels led to changes in the relative levels of AMP, IMP, hypoxanthine, and xanthine, which are involved in purine metabolism. AMP is a precursor of IMP, and IMP degrades into xanthine and hypoxanthine (19, 20). AMP and IMP present an umami taste (21), and hypoxanthine and xanthine are the sources of bitterness (22). Citrulline is one of the primary metabolites of arginine, and AMP is the product of citrulline catabolism (23). Our results suggested that excessive arginine enhanced hypoxanthine synthesis and deficient arginine increased xanthine synthesis, and changing trend of muscular AMP content was similar to muscular citrulline content. The adenosine content increased, whereas IMP synthesis decreased with the increasing dietary arginine levels. The above results may indicate that a diet with supplemented arginine led to the redirection of AMP deamination (24), which elevated citrulline content may cause. The non-targeted metabolic analysis indicated that dietary arginine levels affected the production of bitter substances. Inosine is the primary degradation product of IMP, which is related to bitter taste (25). Targeted metabolic analysis revealed that arginine deficiency enhanced muscular inosine content.

Subsequently, we further explored the effect of arginine levels on muscular FAAs related to flavor in crustaceans (26). Aspartate and glutamate are related to umami taste; alanine, glycine, and serine possess a sweet taste, while leucine and isoleucine take part in the Maillard reaction to synthesize aroma compounds (27). In the study, the contents of aspartate and glutamate increased with the increase of dietary arginine. Meanwhile, the MA group had the highest contents of alanine and serine and total flavor amino acids. These results suggest that an optimal arginine level improved the flavor of the flesh of *L. vannamei* farmed in freshwater. The flavor is one of the crucial factors for consumers in evaluating flesh quality (28). These results indicated that dietary arginine should be appropriate to reduce the production of bitter substances and increase the umami taste of *L. vannamei*.

Our previous study found that dietary supplementation creatine improved *L. vannamei* flesh quality cultured in freshwater (5). Creatine is an essential metabolite of arginine (29). However, our study found that the creatine content of hepatopancreas and muscle was not affected by adding dietary arginine. This result indicated that only increasing the concentration of exogenous arginine cannot bring an increasing endogenous creatine synthesis in muscle and hepatopancreas. Creatine synthesis is involved a complicated process. It requires not only arginine but also glycine and methionine and related enzymes, including L-arginine-glycine amidinotransferase (AGAT), methionine-adenosyltransferase (MAT), and guanidinoacetate-methyltransferase (GAMT) (24). The current study showed that dietary arginine did not affect free methionine content but decreased free glycine content, which may explain unaltered muscular creatine content. Creatine is forming in liver and then is mainly transported skeletal muscle. An important metabolite of creatine is creatinine (30). In our study, dietary arginine levels had no significant effect on muscular creatine content. Still, urea nitrogen content in serum and creatinine in hepatopancreas and muscle were increased with the increasing dietary arginine level and reached the highest values in the HA group. These results indicated that surplus arginine might lead to hepatopancreas disruption of *L. vannamei*. This result explained the decrease in survival of *L. vannamei* caused by excess arginine in this study.

Consumers prefer firmer aquatic products (31). The MA and HA diets improved textural properties in this study, including hardness and chewiness. Collagen content positively correlates with muscular hardness. Ornithine is an essential metabolite of arginine, and it is a precursor for collagen synthesis (32). In this study, muscular ornithine content increased with dietary arginine levels. Alkaline-soluble collagen includes the degraded collagen and the newly synthesized collagen molecules, and alkaline-insoluble collagen is related to the tensile strength of muscle, which contributes to the increase of muscular hardness (33). Our study found that arginine supplementation improved muscular hardness by increasing the content of alkaline-insoluble collagen. Similar results were found in grass carp (8). This result indicated that the increase in collagen content is due to an increase in ornithine content.

Meanwhile, glutamate is one of the primary metabolites of arginine metabolism. Dietary glutamate improved the muscle hardness of Atlantic salmon (34). In this study, shrimps fed the MA and HA diets had significantly higher hydrolyzed glutamate and free glutamate contents in muscle than those fed the LA diet, suggesting that increased muscular hardness may be partly linked to dietary arginine increased muscular glutamate content.

Previous studies indicated that muscular hardness positively correlates with fiber density (35, 36). In addition, smaller myofiber diameter and higher myofiber density reflected better flesh quality (37). In the present study, the myofiber density in the MA group was significantly higher than that in other treatments. On the other hand, the enhancement of flesh hardness in *L. vannamei* by arginine may link to the myofiber diameter. Myofiber diameter (<20 μm) suggests an active hyperplastic growth process during the developmental stage (38). In the current study, the MA group showed a higher frequency of fibers diameter (<20 μm) and a lower frequency of fibers diameter (>50 μm) compared to the LA group, suggesting that optimum arginine level might improve the myofiber hyperplasia and skeletal muscle development (39). In this study, shrimps fed with MA diet and HA diets had longer sarcomere lengths. Sarcomeres consist of alternating light (I-band) and dark (A-band) bands, and longer sarcomeres decrease soluble protein loss and increase water holding capacity (40). This study indicated that arginine might prevent soluble protein loss and improve cooking flesh quality. In addition, sarcomere length is related to myofiber types (41). Myosin heavy chain gene family (MyHCs), a multi-gene family in vertebrate genomes, is closely related to the regulation of myofiber types (42). There are 13 muscle-type myosin genes found in *L. vannamei*, and five MyHCs were chosen to assess the relationship between arginine supplementation and myofiber type. For the first time, the current study showed that dietary arginine down-regulated the mRNA expressions of fast fiber *MyHCs* (*sMyHC1*, *sMyHC2*, and *sMyHC6a*) and up-regulated slow fiber MyHCs (*sMyHC5* and *sMyHC15*) in *L. vannamei*. Slow fibers have longer sarcomeres and shorter diameters than that fast fibers (41). These results are consistent with the trends in sarcomere length and myofiber diameter measured in this study.

## Conclusion

Dietary optimal arginine (21.82 g kg^–1^) improved muscle hardness by increasing the proportion of myofibers (<20 μm), myofiber density, sarcomere lengths, ornithine content and promoting fast myofibers conversion to slow myofibers. The dietary levels of arginine increased the contents of muscular umami amino acids (free aspartate and glutamate) and affected the purine metabolic pathway by reducing the content of hypoxanthine, xanthine, and inosine (bitter substance), thereby improving the flavor of the flesh. Additionally, the elevation of the protein-moisture ratio and hydrolyzed amino acids content, which improved the nutritional values of muscle. Finally, ornithine, citrulline, and glutamate were identified as critical metabolites in affecting flesh quality after arginine treatments. Whether these metabolites are more effective than arginine in improving shrimp flesh quality requires further study.

## Data availability statement

The original contributions presented in this study are included in the article/Supplementary material, further inquiries can be directed to the corresponding author.

## Ethics statement

The animal study was reviewed and approved by Pacific white shrimps (*Litopenaeus vannamei*) are widely cultivated in China and are not listed as endangered or protected species. All animal care and use procedures were approved by the Institutional Animal Care and Use Committee of Yangtze River Fisheries Research Institute (according to YFI 2018-40 of July 20, 2018). Pacific white shrimps were anesthetized with 30 mg eugenol L^–1^ water to minimum suffering before being assigned to cages and were anesthetized death with 60 mg eugenol L^–1^ water when sampling muscle and hepatopancreas in this experiment. Written informed consent was obtained from the owners for the participation of their animals in this study.

## Author contributions

JT and HW designed the research. ML, MW, LY, MJ, and XL conducted the experiments and analyzed the data. ML, FH, and JT wrote this manuscript. All authors have read and approved the final manuscript.
