# Supplementary material for: Role of arginine supplementation on muscular metabolism and flesh quality of Pacific white shrimp (Litopenaeus vannamei) reared in freshwater
Source: Front Nutr. 2022 Aug 31;9:980188. doi: 10.3389/fnut.2022.980188 (PMC9473507; doi:10.3389/fnut.2022.980188)
Supplement: Supplementary file 1 [file Data_Sheet_1.docx]

| Table S1. Formulation and proximate composition of the experimental diets (g kg^-1^). | | | |
| --- | --- | --- | --- |
| Ingredients (g kg^-1^) | Diets | | |
|  | LA | MA | HA |
| Corn meal | 200.00 | 200.00 | 200.00 |
| Fish meal | 200.00 | 200.00 | 200.00 |
| Wheat gluten meal | 70.00 | 70.00 | 70.00 |
| Essential amino acid^1^ | 30.00 | 30.00 | 30.00 |
| Nonessential amino acid^2^ | 40.00 | 30.00 | 20.00 |
| Arginine | 0.00 | 10.00 | 20.00 |
| Corn starch | 300.00 | 300.00 | 300.00 |
| Sodium alginate | 23.00 | 23.00 | 23.00 |
| Eclosion hormone | 1.50 | 1.50 | 1.50 |
| Fish oil | 15.00 | 15.00 | 15.00 |
| Soybean oil | 15.00 | 15.00 | 15.00 |
| Soybean phospholipid powder | 10.00 | 10.00 | 10.00 |
| Astaxanthin | 1.00 | 1.00 | 1.00 |
| Cholesterol | 5.00 | 5.00 | 5.00 |
| Cellulose | 46.00 | 46.00 | 46.00 |
| Vitamin premix^3^ | 10.00 | 10.00 | 10.00 |
| Mineral premix^4^ | 10.00 | 10.00 | 10.00 |
| CaH_2_PO_4_ | 20.00 | 20.00 | 20.00 |
| Vitamin C | 1.00 | 1.00 | 1.00 |
| Choline chloride | 2.50 | 2.50 | 2.50 |
| Proximate composition (g kg^-1^) | | | |
| Dry matter | 946.00 | 946.11 | 946.43 |
| Crude protein | 424.68 | 424.13 | 424.05 |
| Crude lipid | 70.04 | 70.34 | 70.74 |
| Ash | 64.30 | 65.71 | 64.41 |
| Arginine | 10.15 | 21.82 | 32.46 |
| ^1^ Essential amino acids (g kg^-1^ diet): methionine, 2; threonine, 3; phenylalanine, 12; lysine, 10; valine, 3.  ^2^ Non-essential amino acids (g kg^-1^ diet): aspartate, 9; glutamate, 13.5; glycine, 13.5.  ^3^ Vitamin premix contained (g kg^−1^ mixture): thiamin hydrochloride, 6 g; riboflavin, 5 g; pyridoxine hydrochloride, 6 g; nicotic acid, 10 g; inositol, 119 g; calcium pantothenate, 12 g; biotin, 0.1 g; folic acid, 1 g; cobalamine, 0.02 g; menadione, 6 g; retinyl acetate, 1 g; cholecalciferol, 0.4 g; alpha-tocopherol acetate 10 g. All ingredients were diluted with alpha-cellulose to 1 kg.  ^4^ Mineral premix contained (g kg^−1^ mixture): ferric citrate, 7.36 g; potassium iodate, 0.02 g; ZnSO_4_•7H_2_O, 14.14 g; MnSO_4_•H_2_O, 6.22 g; CuSO_4_•5H_2_O, 9.28 g; Na_2_SeO_3_, 0.03 g; CoCl_2_•6H_2_O, 2.03 g; MgSO_4_•7H_2_O, 500 g; sodium chloride, 80 g; potassium chloride, 50 g; calcium pantothenate, 75 g; All ingredients were diluted with alpha-cellulose to 1 kg. | | | |

| Table S2. Amino acids contents of experimental diets (g kg^-1^). | | | |
| --- | --- | --- | --- |
| Indexes | Diets | | |
|  | LA | MA | HA |
| Essential amino acids | | | |
| Arg | 10.15 | 21.82 | 32.46 |
| His | 5.89 | 5.54 | 5.61 |
| Ile | 10.17 | 9.49 | 10.60 |
| Leu | 28.73 | 27.32 | 30.87 |
| Lys | 18.08 | 17.35 | 18.57 |
| Met | 7.73 | 7.98 | 8.92 |
| Phe | 22.58 | 24.05 | 25.47 |
| Thr | 12.05 | 11.30 | 12.70 |
| Val | 13.13 | 14.45 | 14.99 |
| Non-essential amino acids | | |  |
| Ala | 15.63 | 13.69 | 15.28 |
| Asp | 25.13 | 21.47 | 20.27 |
| Cys | 16.00 | 23.68 | 15.06 |
| Glu | 67.13 | 58.12 | 40.57 |
| Gly | 22.27 | 18.44 | 16.11 |
| Ser | 11.55 | 11.74 | 13.71 |
| Pro | 22.24 | 21.74 | 27.03 |
| Tyr | 11.63 | 12.44 | 12.85 |
| ΣEAA | 128.51 | 139.30 | 160.19 |
| ΣNEAA | 191.58 | 181.32 | 160.88 |
| TAAs | 320.09 | 320.63 | 321.07 |
| ΣEAA: Total essential amino acids.  ΣNEAA: Total non-essential amino acids.  TAAs: Total amino acids. | | | |

| **Table S3**  Nucleotide sequences of primers and cycling conditions used for PCR amplification. | | | | | |
| --- | --- | --- | --- | --- | --- |
| Gene | Accession no. |  | Primer sequence(5' to 3') | Amplification size | Tm |
| REFERENCE GENE | | | | | |
| *EF1α_1_* | XM_027373349.1 | Forward | TGCTCTGGACAACATCGAGC | 101 | 60 |
|  |  | Reverse | CGGGCACTGTTCCAATACCT |  |  |
| FAST FIBER GENE | | | | | |
| *sMYCH1* | AB758443 | Forward | CGGTGCCTCTGAGAAGAAAG | 119 | 58 |
|  |  | Reverse | AGGAGTTGTCGTTACGGGTG |  |  |
| *sMYCH2* | AB758444.1 | Forward | CGATATTTACGACTACCGCTACG | 192 | 59 |
|  |  | Reverse | CGACCCCTCTGCTTGAACTT |  |  |
| *sMYCH6a* | AB759104.1 | Forward | ATCCGAACTTGCTGATGCC | 207 | 60 |
|  |  | Reverse | TCAGCACGGAGTTCGTCAGC |  |  |
| SLOW FIBER GENE | | | | | |
| *sMYCH5* | AB759100.1 | Forward | ATGCTCAACGAAGCCAGACA | 193 | 60 |
|  |  | Reverse | CCTTCATCGCATTTGTTTCG |  |  |
| *sMYCH15* | LVANscaffold_903 446522: 459841 | Forward | GCAACTACGCCACCGAACAC | 132 | 60 |
|  |  | Reverse | CCTCACCGATCTGGTCCATCAA |  |  |
